# Spectral, cepstral, and short-term acoustic measures in women with and without laryngeal and vocal alterations

**DOI:** 10.1590/2317-1782/e20250344en

**Published:** 2026-06-15

**Authors:** Amanda Mariane Paulino Gualberto, Fabiana Andrade Penido, Ana Cristina Côrtes Gama

**Affiliations:** 1 Curso de graduação em Fonoaudiologia, Faculdade de Medicina, Universidade Federal de Minas Gerais – UFMG - Belo Horizonte (MG), Brasil.; 2 Superintendência Central de Perícia Médica e Saúde Ocupacional do Estado de Minas Gerais - Belo Horizonte (MG), Brasil.; 3 Departamento de Fonoaudiologia, Universidade Federal de Minas Gerais – UFMG - Belo Horizonte (MG), Brasil.

**Keywords:** Acoustic Analysis of Speech, Voice, Laryngeal Disorders, Voice Disorders, Voice Quality

## Abstract

**Purpose:**

To compare acoustic voice measures of women with and without laryngeal and vocal alterations.

**Methods:**

Cross-sectional, analytical, observational study with 69 female teachers, divided into four groups: 21 with laryngeal alterations (LAG), 48 without alterations (NAG), 45 with vocal deviations (VDG), and 24 without deviations (NDG). The groups were defined by otolaryngological evaluation (videolaryngoscopy or videolaryngostroboscopy) and by auditory-perceptual evaluation of the grade of hoarseness. Participants who smoked, were pregnant, menstruating, or had allergies or the flu were excluded. Voice and speech samples (3 central seconds of the vowel [a] and counting from 1 to 11) were recorded and edited using Praat. The acoustic measures jitter, shimmer, harmonic-to-noise ratio (HNR), glottal-to-noise excitation (GNE), smoothed cepstral peak prominence (CPPS), spectral tilt, high-frequency noise (HF noise), and the difference between the first and second harmonics (H1-H2) were extracted from the VOXplot. Statistical analysis was performed using the t-test, Mann-Whitney test, and Hedges' g test.

**Results:**

Women with laryngeal and vocal alterations had lower GNE, HNR, CPPS, H1-H2, and HF noise values and higher jitter, shimmer, and spectral tilt values. CPPS, spectral tilt (vowel), and H1-H2 (vowel and speech) were not different between the groups with and without laryngeal alterations. CPPS (vowel) and spectral tilt (vowel and speech) were likewise not different between the groups with and without vocal deviation.

**Conclusion:**

The acoustic measures responded similarly to the presence of laryngeal and vocal alterations, reinforcing the combined influence of structural and functional factors on the quality of the vocal signal.

## INTRODUCTION

The human voice is a primary tool for communication, reflecting the speaker’s individual and personality characteristics^(1)^. It also plays a crucial role in the quality of communication, influencing how listeners perceive and understand the message^(2,3)^. However, various factors can affect the voice, including anatomical and physiological changes in the larynx, which can cause noticeable differences in its acoustic properties^(2)^. Understanding the acoustic characteristics of the voice is essential for assessing vocal quality^(4)^.

There is a variety of methodologies for voice assessment, including acoustic analysis, auditory-perceptual evaluation, aerodynamic assessment, visual laryngeal examination, and vocal self-assessment^(5)^. Each of these evaluative approaches plays an important role in multidimensional vocal assessment, providing a comprehensive view of the characteristics of vocal production^(6)^. The aerodynamic myoelastic theory is the most widely accepted to explain the phonation process, providing important information about the vibratory mechanism of the vocal folds. This becomes especially relevant due to the association of many vocal disorders with abnormal vocal fold oscillation patterns^(7)^.

Assessing voice quality poses a significant challenge. Subjective judgment, considered the gold standard, is based on auditory perception and is influenced by various factors, ranging from socioeconomic and cultural elements to personal tastes, despite its great importance and use in vocal clinical practice^(1)^. In this context, acoustic voice assessment complements auditory-perceptual evaluation and has gained prominence in vocal clinical practice for being non-invasive and objective^(5)^. This is evidenced by the continuous development of various acoustic parameters, whose purpose is to measure the vocal signal and provide relevant clinical information in the assessment of dysphonia^(6)^.

The literature^(8,9)^ describes several acoustic measures, such as **1) the spectra and coefficients obtained by linear and Fourier prediction** ^(9,10)^, including cepstral measures, cepstral peak prominence (CPP), and cepstral peak prominence-smoothed (CPPS), which highlight the prominence of harmonics in relation to the noise present in the voice signal^(8)^; the spectral tilt, characterized as the spectral decline obtained from the long-term average spectrum (LTAS) of the vocal sound radiated by the lips, reflecting both the properties of the glottal source and the formant characteristics of the vocal tract^(11)^; the high-frequency noise (HF noise), which measures the difference between the relative acoustic energy between 0 and 6 kHz and between 6 and 10 kHz^(11)^; the H1-H2, which analyzes the difference in amplitude between the first and the second harmonic^(12)^; the harmonic-to-noise ratio (HNR), which represents the relationship between periodic energy and noise energy^(13)^; and the glottal-to-noise excitation ratio (GNE), which quantifies the additive noise present in the voice signal^(14)^; **2) short-term amplitude perturbation measures** ^(15)^, which quantify cycle-to-cycle variations in the amplitude of the voice signal, such as shimmer; and **3) short-term frequency perturbation measures** ^(15)^, which quantify cycle-to-cycle variations in the fundamental frequency of the voice signal, such as jitter.

These acoustic measures play a fundamental role in vocal characterization, providing objective and robust information that can be used to assess different dysphonia conditions and monitor the evolution of vocal treatment^(16)^.

Understanding how laryngeal alterations affect the acoustic characteristics of the voice is crucial for discerning vocal nuances in healthy contexts and in dysphonia, since these alterations can generate a lack of vocal harmony, impede natural voice production, and alter the quality of vocal emission^(1,17)^. The reliable and valid application of objective acoustic analysis in research or clinical practice requires attention to specific requirements, such as the quality of the sound card, type of microphone, acoustic analysis program, and examination conditions, to guarantee a highly precise and reliable vocal analysis^(18)^ and the possibility of using different acoustic parameters that evaluate vocal signals in the time, frequency, amplitude, and frequency domains^(18)^.

Another aspect to consider is that acoustic analysis focused on the evaluation of sustained vowels has been a common practice in both research and clinical practice. However, the literature highlights the importance of enriching this approach by incorporating not only the sustained vowel task but also the analysis of connected speech^(9)^, when possible. This expansion is based on the growing understanding that combining acoustic analysis in both vocal tasks contributes significantly to the ecological validity of this type of vocal investigation^(17)^. The inclusion of speech tasks adds a more realistic element to the assessment, reflecting more faithfully the natural conditions of voice use. This, in turn, provides more robust and comprehensive estimates, increasing the diagnostic accuracy of the parameters analyzed^(9,17)^.

Thus, this research aimed to compare different acoustic measures in sustained vowel and connected speech tasks of the voices of women with and without laryngeal and vocal alterations. Through this comparative analysis, the research expects to enrich the knowledge about the acoustic characteristics of female voices in different laryngeal and vocal contexts, with and without alteration, and thus contribute to improving the assessment and treatment of dysphonia.

Considering the importance of the voice as a fundamental tool in various professions, from teaching to singing, and the fact that laryngeal alterations can negatively impact vocal quality and the professional and personal lives of dysphonic women^(19)^, it is imperative to understand the acoustic variations associated with these laryngeal and vocal conditions. This understanding is crucial to improve the assessment and treatment of dysphonia in vocal clinical practice^(20)^.

## METHODS

This is a cross-sectional, analytical, observational study approved by the research ethics committee of the Federal University of Minas Gerais (UFMG) under approval number 39351920.2.0000.5149.

All study participants were informed about the research objectives and procedures and signed an informed consent form.

The study included 69 women, teachers from the Minas Gerais state school system, aged 22 to 44 years (mean = 36.81; SD = 6.64), divided into two groups: 21 women with laryngeal alteration (LAG) and 48 women without laryngeal alteration (NAG). These groups were defined based on the otolaryngological evaluation using videolaryngoscopy or videolaryngostroboscopy.

The teachers’ voices underwent expert speech-language-hearing evaluation at the Central Superintendence of Medical Expertise and Occupational Health (SCPMSO) of the State of Minas Gerais, and they presented a laryngeal examination, performed by an otolaryngologist of their choice.

The sample size corresponds to the total number of teachers who simultaneously met the established clinical, vocal, and laryngeal criteria, constituting a convenience sample, a common characteristic in observational designs.

The NAG inclusion criterion was the absence of laryngeal or glottal closure alterations, described by otolaryngologists in the laryngeal examination report. Hence, this group included teachers who presented a description of complete glottal closure or a posterior triangular chink, considered physiological in women^(21)^.

LAG included teachers whose otolaryngological report indicated insufficient glottal closure and alterations in the edges of the vocal folds. The examinations were performed by different otolaryngologists. Then, two speech-language-hearing pathologists specializing in voice analyzed the recordings independently to verify the presence of the alterations described in the report. The study only included cases in which the otolaryngological diagnosis agreed with the consensual analysis of the speech-language-hearing pathologists – i.e., when both professionals verified the laryngeal alterations in the recordings. Two of the 23 recordings analyzed were excluded for not presenting this agreement, leaving only the cases with consensus in the evaluation in the final sample.

The exclusion criteria for both study groups were being a smoker, pregnant, or in the menstrual period, and having an allergic process or flu-like symptoms.

The voices of the 69 teachers were recorded in a quiet room at SCPMSO with an ambient noise below 40 dB SPL, measured using a DEC brand decibel meter – model 5010.

The voice and speech samples were recorded on an Apple MacBook Air computer with a professional sound card using a unidirectional condenser microphone, an Andrea Pureaudio USB adapter, and a Karsect HT-9 unidirectional condenser microphone, mounted on the head 10 cm away from the corner of the participants' mouths. The samples were digitized at a sampling rate of 44.1 kHz and amplitude resolution of 16 bits, in .wav format, using Praat software (version 6.1.47)^(5)^.

The teachers stood and were instructed to emit a sustained vowel [a:] and count from 1 to 20, at usual frequency and intensity, for the extraction of acoustic measures.

**Figure 1:**
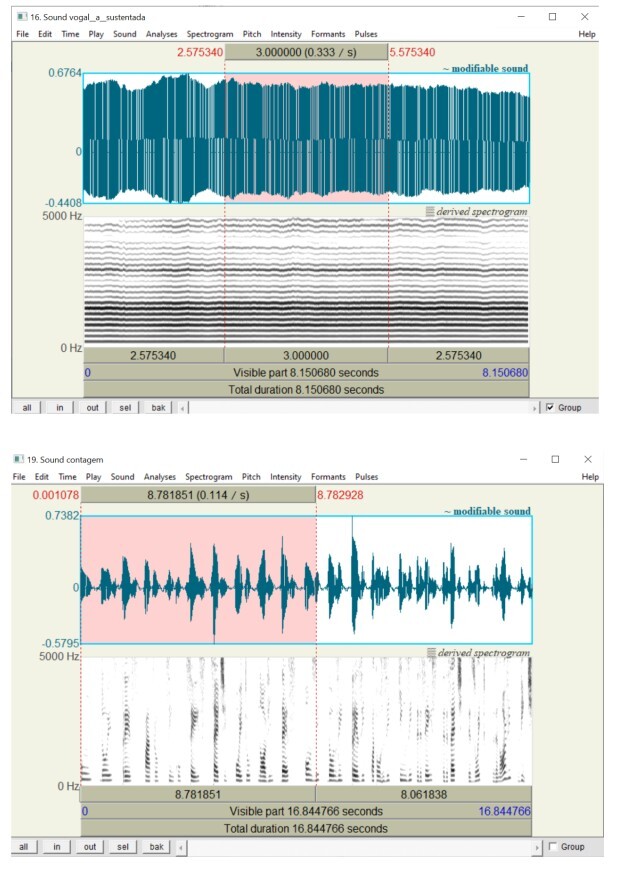
Praat screenshots showing the selection of the central 3 seconds of the vowel [a] (above) and the count from 1 to 11 (below)

After recording the samples, the voice and speech files were edited in Praat software version 6.1.47. The central 3 s of the vowel [a:] at usual frequency and intensity were selected manually, as they correspond to the most stable portion of the vocal signal. In addition, counting numbers from 1 to 11 was used as a connected speech task, which is widely used in Brazilian speech-language-hearing clinical practice, presenting a better correlation between perceptual and acoustic measures^(18)^ (Figure 1).

**Figure 2:**
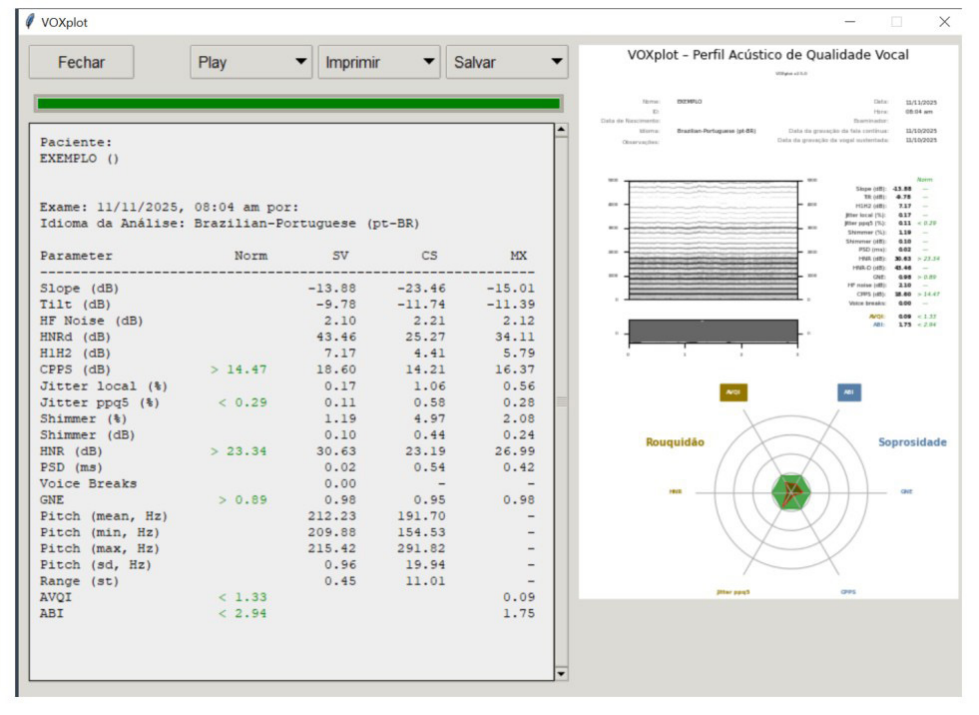
VOXplot (version 2.5.0) screenshot of the analysis of vocal quality and acoustic measures

The voice and speech files, recorded and edited in Praat, were named sv (sustained vowel) and cs (connected speech), respectively, and loaded into VOXplot version 2.5.0^(11)^ for the extraction of acoustic measures (Figure 2). VOXplot calculates the acoustic measures of the vocal and counting tasks, displays the oscillogram and narrowband spectrogram of the vowel [a], and shows a graph illustrating the values ​​of six acoustic dimensions used to assess hoarseness and breathiness in the voice, namely: HNR, jitter (ppq5), GNE, CPPS, Acoustic Voice Quality Index (AVQI), and Acoustic Breathiness Index (ABI)^(22)^ (Figure 2).

The acoustic measures of jitter (%), shimmer (%), HNR (dB), and GNE were obtained from the vowel, while spectral tilt (dB), HF noise (dB), H1-H2 (dB), and CPPS (dB) were obtained from the vocal and number counting tasks.

Three speech-language-hearing pathologists with more than 10 years of experience in this type of analysis performed the auditory-perceptual evaluation of the voice and speech samples independently to characterize the groups regarding vocal quality. The samples were classified according to the GRBASI scale^(23)^, using the G parameter, referring to the overall grade of hoarseness. GRBASI is a 4-point Likert scale, where 0 = no vocal alteration, 1 = slight vocal alteration, 2 = moderate vocal alteration, and 3 = severe vocal alteration.

The statistical analysis of the data was performed using the MINITAB statistical program, version 17. First, a descriptive analysis of the data was performed with measures of central tendency and dispersion. Then, the Anderson-Darling test was used to verify the normality of the sample. The t-test was used to compare HNR (dB), CPPS (dB), spectral tilt, HF noise (dB), and H1-H2 (dB) between the LAG and NAG, while the non-parametric Mann-Whitney test was used for the jitter, shimmer, and GNE measures. The confidence level was set at 95%.

The effect size for each acoustic measure was calculated using Hedges’ g, which is suitable for comparing two independent groups with moderate sample sizes and distinct because it corrects Cohen’s d estimate for small samples. The effect sizes were interpreted according to conventional criteria: small (≈ 0.2), moderate (≈ 0.5), and large (≥ 0.8)^(24)^.

## RESULTS

The LAG consisted of 21 women who received an otolaryngological diagnosis of nodules (n = 16; 76.2%), polyps (n = 2; 9.5%), cysts (n = 1; 4.8%), and hourglass and mid-posterior triangular glottal chinks (n = 2; 9.5%).

The three expert speech-language-hearing pathologists classified the voice and speech samples of the groups with and without laryngeal alteration, considering the G of the GRBASI scale. According to the classification of at least two of the three expert speech-language-hearing pathologists, the NAG consisted of 24 (50%) women with neutral vocal quality (G0) and 24 (50%) with slight deviation (G1). The LAG consisted of 10 (47.61%) teachers with slight vocal deviation, seven (33.33%) with moderate vocal deviation, and four (19.06%) with severe vocal deviation.

Table 1 shows the means, standard deviation, and comparison of acoustic measures between NAG and LAG.

**Table 1:** Comparison of acoustic measurements between groups of women with and without laryngeal alterations

**ACOUSTIC MEASURES**	**GROUP**	**MEAN**	**SD**	**p-value**	**EFFECT SIZE**
**Jitter (%) (sv)**	NAG	0.35	0.21	**0.017** ^#^	-0.67
	LAG	0.54	0.36		
**Shimmer (%) (sv)**	NAG	2.66	1.55	**< 0.001#**	-1.08
	LAG	4.71	2.26		
**HNR (dB) (sv)**	NAG	24.66	4.26	< 0.000*	1.21
	LAG	19.44	4.26		
**GNE (sv)**	NAG	0.93	0.07	**0.0094#**	0.63
	LAG	0.88	0.09		
**CPPS (sv)**	NAG	15.61	2.23	0.063*	0.53
	LAG	14.29	2.75		
**CPPS (cs)**	NAG	12.87	1.38	0.002*	1.01
	LAG	11.11	2.12		
**H1-H2 (dB) (sv)**	NAG	4.33	5.46	0.277*	0.29
	LAG	2.7	5.71		
**H1-H2 (dB) (cs)**	NAG	1.74	2.82	0.062*	0.57
	LAG	-0.45	4.81		
**Spectral tilt (dB) (sv)**	NAG	-10.59	1.17	0.26*	-0.31
	LAG	-10.2	1.35		
**Spectral tilt (dB) (cs)**	NAG	-11.58	0.99	0.009*	-0.8
	LAG	-10.61	1.44		
**HF Noise (dB) (sv)**	NAG	2.19	0.2	< 0.000*	1.09
	LAG	1.96	0.22		
**HF Noise (dB) (cs)**	NAG	2.26	0.19	0.005*	0.85
	LAG	2.07	0.26		

* t-test;

# Mann-Whitney Test

**Caption:** HNR: harmonic-to-noise ratio; GNE: glottal-to-noise excitation; CPPS: cepstral peak prominence-smoothed; H1-H2: H1-H2 amplitude difference; spectral tilt: spectral decline; HF noise: high-frequency noise; sv: sustained vowel; cs: connected speech; dB: decibel; Hz: Hertz; SD: standard deviation; NAG: group without laryngeal alteration; LAG: group with laryngeal alteration

The comparative analysis between the groups showed that women with laryngeal alterations (LAG) have lower values, with a statistically significant difference, in GNE (vowel), HNR (vowel), CPPS (speech), and HF noise (vowel and speech). Higher values ​​were also observed for jitter (vowel), shimmer (vowel), and spectral tilt (speech) in LAG, with a significant difference between the groups. LAG had lower values ​​for H1-H2 (vowel and speech) and CPPS (vowel), with no significant difference between the groups. LAG also had a higher spectral tilt value in the vowel task than NAG, without a significant difference.

Large effect sizes were observed for shimmer and HNR (vowel), CPPS and spectral tilt (speech), and HF noise (vowel and speech). Moderate effects were found for jitter, GNE, and CPPS (vowel), and H1-H2 (speech). These findings indicate that, in addition to being statistically significant, several of these differences have sufficient magnitude to be clinically relevant.

Table 2 compares acoustic measures between the groups of women without vocal deviations (NDG) (n = 24) and those with vocal deviations (VDG) (n = 45), encompassing women with mild (n = 34), moderate (n = 7), and severe (n = 4) deviations.

**Table 2:** Comparison of acoustic measures between groups of women with and without vocal deviation

**ACOUSTIC MEASURES**	**GROUP**	**MEAN**	**SD**	**p-value**	**EFFECT SIZE**
**Jitter (%) (sv)**	NDG	0.27	0.09	**0.0017#**	-0.98
	VDG	0.48	0.31		
**Shimmer (%) (sv)**	NDG	2.39	1.54	**0.0025** ^#^	-0.76
	VDG	3.76	2.09		
**HNR (dB) (sv)**	NDG	26.12	4.56	**< 0.000** *	1.04
	VDG	21.44	4.24		
**GNE (sv)**	NDG	0.94	0.04	**0.03#**	0.6
	VDG	0.9	0.09		
**CPPS (sv)**	NDG	15.86	2.34	0.104*	0.41
	VDG	14.86	2.46		
**CPPS (cs)**	NDG	12.99	1.45	0.018*	0.6
	VDG	11.99	1.91		
**H1-H2 (dB) (sv)**	NDG	5.99	4.13	0.009*	0.66
	VDG	2.68	5.9		
**H1-H2 (dB) (cs)**	NDG	2.52	2.78	0.008*	0.67
	VDG	0.3	3.85		
**Spectral tilt (dB) (sv)**	NDG	-10.65	1.02	0.358*	-0.23
	VDG	-10.38	1.33		
**Spectral tilt (dB) (cs)**	NDG	-11.61	1.11	0.103*	-0.41
	VDG	-11.12	1.26		
**HF Noise (dB) (sv)**	NDG	2.23	0.19	0.002*	0.81
	VDG	2.06	0.23		
**HF Noise (dB) (cs)**	NDG	2.29	0.19	0.016*	0.62
	VDG	2.16	0.23		

* t-test;

# Mann-Whitney Test

**Caption:** HNR: harmonic-to-noise ratio; GNE: glottal-to-noise excitation; CPPS: cepstral peak prominence-smoothed; H1-H2: H1-H2 amplitude difference; spectral tilt: spectral decline; HF noise: high-frequency noise; sv: sustained vowel; cs: connected speech; dB: decibel; Hz: Hertz; SD: standard deviation; NDG: group without vocal deviation; VDG: group with vocal deviation.

The groups were significantly different in all acoustic measures, except for CPPS (vowel) and spectral tilt (vowel and speech).

The group of women with vocal deviations had significantly lower GNE (vowel), HNR (vowel), CPPS (speech), and HF noise (vowel and speech) values, a pattern similar to that observed in the comparison between the groups with and without laryngeal alterations. The H1-H2 was also statistically significantly different between the groups in the vowel and speech tasks, with lower values in the group with vocal deviations. This fact was not verified in the comparison between the groups with and without laryngeal alterations.

The group with vocal deviations had higher jitter and shimmer values, with a statistically significant difference.

Large effect sizes were observed for jitter, HNR, and HF noise (vowel), and moderate effects for shimmer and GNE (vowel), CPPS (speech), and H1-H2 in both tasks. Spectral tilt parameters showed small effects, even when accompanied by statistically significant differences.

## DISCUSSION

The voice is a fundamental feature in human communication and reflects the health and functioning of the larynx^(3)^. Laryngeal disorders can significantly impact vocal quality, requiring precise and sensitive diagnostic tools for their detection and monitoring^(1)^.

Acoustic voice analysis is a valuable resource in this scenario, as it allows for an objective and quantitative assessment of vocal characteristics^(9)^. This research investigated the behavior of acoustic measures that provide information regarding the glottal source, such as jitter (%), shimmer (%), HNR, GNE, spectral tilt, CPPS, and H1-H2, in sustained vowel and connected speech tasks in the voices of women with and without laryngeal and vocal alterations.

Regarding the frequency and amplitude disturbance measures, Jitter (%) and Shimmer (%), it was found that both the group of women with laryngeal alterations and the group with vocal deviations presented higher values ​​of these measures in the vowel [a] than the groups without laryngeal alterations and without vocal deviations, as expected (Tables 1 and 2). Moreover, these measures were significantly different between the groups with and without laryngeal and vocal alterations. This fact indicates that the laryngeal alterations presented by the women in LAG interfered with the periodicity of the frequency and amplitude vibration of the vocal folds in the short term, affecting their vocal quality.

There are several ways to obtain jitter and shimmer; this study analyzed the values ​​of local jitter and shimmer (%). Local jitter (%) relates to the absolute average difference between successive periods, divided by the average period. Local shimmer (%) refers to the absolute average difference between the amplitudes of successive periods divided by the average amplitude^(13)^.

Studies show that shimmer changes mainly in situations of reduced glottal resistance (e.g., incomplete glottal closure) and is also influenced by mass lesions, even small ones^(1,4)^. Jitter changes mainly with the lack of control of vocal fold vibration^(25)^.

A study with women with vocal complaints extracted jitter, shimmer, and other acoustic measures and verified an increase in their values as vocal quality worsened^(26)^. In this study, as mentioned, vocal deviation was associated with an increase in the values ​​of these measures and with laryngeal alterations.

Shimmer was also significantly different in another study with women with and without vocal nodules^(10)^. The jitter and shimmer values were 0.87% and 6.91% among those with vocal nodules and 0.28% and 3.31% among those without nodules, with the vowel [a]. Similarly, jitter and shimmer values in the present study ​​were lower in NAG than in LAG (LAG: jitter = 0.54%; shimmer = 4.71%; NAG: jitter = 0.35%; shimmer = 2.66%), with LAG showing deviated values. VOXplot reference values ​​for jitter (%) and shimmer (%) for healthy voices are < 0.5% and < 3%, respectively^(11)^.

The study results for jitter and shimmer are also consistent with another study that found a significant difference in both measures in control voice signals and signals from laryngeal alterations, such as polyps on the vocal folds^(27)^.

Regarding HNR, another short-term acoustic measure, significant differences were found between the groups with and without laryngeal and vocal alterations. HNR indicates the relationship between the periodic (corresponding to vocal fold vibration) and aperiodic (glottal noise) components of the voice signal, providing information related to phonation efficiency. Thus, the smaller the harmonic component, the greater the noise component^(28)^. The findings of this study indicate that the laryngeal alterations presented by the women in LAG impacted the efficiency of their phonation. In general, lesions at the edge of the vocal folds result in incomplete glottal closure, with air leakage and airflow turbulence during vocal production^(29)^. Thus, it was expected that women from LAG and VDG would present lower HNR values ​​than women from NAG and NDG, as the study verified (HNR: LAG = 19.44 and NAG = 24.66; VDG = 21.44 and NDG = 26.12). The higher the value of this acoustic measure, the lower the presence of noise in the acoustic signal.

These findings are consistent with studies that investigated HNR to differentiate voices with and without deviations^(30,31)^. Researchers also found a strong correlation between this measure and hoarseness in vocal quality^(11,32)^.

Considered a measure of harmonicity, GNE can be used to quantify the additive noise present in the voice signal and is a robust measure to discriminate breathiness from hoarseness^(11)^ and identify the intensity of vocal deviation^(26)^. Through this measure, it is possible to estimate the amount of energy of the vocal fold vibration and the turbulence caused by vocal deviation, when present. Higher GNE values ​​indicate a greater proportion of glottal energy in relation to the noise energy of the acoustic signal^(33)^. The analysis of GNE found that the groups without laryngeal and vocal alterations (NAG and NDG) had significantly higher values than the groups with alterations (LAG and VDG), as demonstrated in the medians in Tables 1 and 2. A study with women with and without vocal nodules likewise found higher GNE values ​​for those without vocal nodules^(10)^ (GNE = 0.63 and 0.82, respectively).

Another study investigated the most effective acoustic parameters for estimating hoarseness and breathiness in vocal quality, using the emission of the vowel [a] as a task and the VOXplot software. It found that GNE values ​​below 0.91 tend to signal hoarseness, and values ​​below 0.89 indicate breathiness^(11)^. GNE and CPPS were considered the best measures to detect breathiness in vocal quality^(11)^. The present study observed that the groups without laryngeal and vocal alterations had GNE values ​​above the cutoff verified in the aforementioned study (GNE: NAG = 0.93 and NDG = 0.94), indicating adequate glottal efficiency in these groups.

When investigating cepstral measures, which can be extracted both in the sustained vowel and in the connected speech task, it was observed that the CPPS was significantly different between the groups of women with and without laryngeal and vocal alterations, only for the speech sample, with higher CPPS values ​​for the NAG and NDG, as expected (CPPS: NAG = 12.87 and LAG = 11.11; NDG = 12.99 and VDG = 11.99). This indicates that the women in the NAG and NDG had a greater harmonic structure than the women in the groups with laryngeal and vocal alterations.

In line with the present research, a study that investigated cepstral measures in the vowel [a] in individuals with nodules found lower values of this measure in those with laryngeal lesions^(34)^. Another study found that individuals with unaltered voices had higher CPPS values due to a well-defined harmonic structure. In contrast, individuals with vocal deviations had lower values ​​of this measure^(8)^.

CPPS values can vary depending on the task. In general, those obtained from sustained vowels are higher than those from speech tasks, due to greater stability in phonation during vowel emission^(35)^. This study did not observe this trend, nor did it find significant differences between the groups for the vowel [a] emission task. This finding is consistent with a study that investigated acoustic and auditory-perceptual measures in women with and without vocal nodules^(10)^. This fact may be related to the task, since the CPPS extracted from the speech task has greater discriminatory power between normal and altered voice than the vowel CPPS^(35)^.

The short-term measures, jitter, shimmer, and HNR, proved to be sensitive to discrimination between the groups with and without laryngeal and vocal alterations. The literature reveals that short-term measures are more useful for mild to moderate vocal deviations because their extraction ​​depends on comparing the harmonic structure cycle by cycle. Thus, for their use to be viable, the voices cannot present a strongly aperiodic structure^(28)^, a fact verified in the voice samples in the present study, which had only four women with severe vocal deviation.

The analysis of H1-H2 results, which represents the difference in amplitude of the first harmonic in relation to the second, found lower values in LAG and VDG, as verified in previous studies^(36,37)^. The first harmonic is related to the peak f0 amplitude in dB (H1), while the second harmonic refers to the peak amplitude of twice the value of f0 (H2). The relationship between these measures reveals phonatory characteristics of the voice. Moreover, the H1-H2 value is associated with the relative length of the open phase of the glottal oscillation^(38)^. An incomplete or slow glottal closure phase appears to increase the amplitude difference between H1 and H2. Thus, in breathy voices, the amplitude of the first harmonic is expected to be higher than that of the other harmonics. Conversely, when H2 is higher than H1 (producing a negative value), a strained or firmer voice, with greater subglottic pressure, is expected. In a voice without deviations, H1 and H2 values are similar^(39)^.

The groups without laryngeal and vocal alterations in this study had higher H1-H2 values in the vowel and speech tasks than LAG and VDG (Tables 1 and 2). Furthermore, significant differences in this acoustic measure were found only when comparing the groups with and without vocal deviations.

The fact that LAG and VDG had lower H1-H2 values than the groups without alterations (NAG and NDG) was not verified in two studies in Brazilian Portuguese (BP) but was observed in an investigation with women with laryngeal lesions and without vocal deviations^(40)^. One of those in BP analyzed H1-H2 in women with behavioral dysphonia, with and without laryngeal alterations^(41)^, and found higher average values ​​for the group with lesions (H1-H2 = 3.64) than for the group without lesions (H1-H2 = 1.65). Similarly, another study with women with and without vocal nodules identified higher H1-H2 values ​​in the group with lesions (H1-H2 = 7.42) than in the control group (H1-H2 = 4.94)^(10)^.

It should be noted that H1-H2 varies greatly in intersubject comparison^(42)^. The present study and those in BP^(10,41)^ observed this variability, evidenced by the wide dispersion of H1-H2 values ​​around the group means. Future research with a larger sample and greater external validity is needed to understand these results better.

Spectral tilt describes the slope of the LTAS regression line^(11)^. The greater its slope, the lower the value of this measure. The spectral tilt, obtained from the vowel and speech samples, was significantly different only between the groups with and without laryngeal alterations in the speech samples. Moreover, NAG and NDG had lower values ​​of this measure than LAG and VDG, indicating a greater slope of the line in women without laryngeal and vocal alterations.

The results of this study indicate that laryngeal and vocal alterations increased spectral tilt values, leading to a lower slope of the line. This finding may be related to compensatory mechanisms and increased strain during glottal closure in women in LAG, who mostly presented lesions on the edges of the vocal folds.

A study investigated the measures of a multiparametric acoustic index, namely the Acoustic Voice Quality Index (AVQI), in individuals with and without dysphonia secondary to different pathological conditions. It found no differences in spectral tilt between the groups. The researchers concluded that this measure might not help to discriminate pathological conditions when used alone^(11)^.

The literature is scarce in studies that investigated spectral tilt and HF noise alone to differentiate groups with and without laryngeal and vocal alterations. HF noise measures the relative level of high-frequency noise between the energy of 0 to 6 kHz and the energy of 6 to 10 kHz^(11)^. It was observed that this measure was sensitive in both vowel and speech and revealed significant differences between the groups with and without laryngeal and vocal alterations (Tables 1 and 2). LAG and VDG had lower values ​​of this measure for the two tasks investigated than NAG and NDG, as expected. This fact indicates greater noise energy at high frequencies of the spectrum, above 6 kHz, in the presence of laryngeal and vocal alterations.

A study compared the diagnostic validity results of isolated acoustic measures of vocal quality that are stronger for perceiving hoarseness and breathiness using the VOXplot. It found cutoffs for HF noise (vowel) of 2.28 dB and 2.29 dB for the perception of hoarseness and breathiness^(11)^. In the present study, the values ​​of this measure are below the cutoffs established for the perception of breathiness and hoarseness in the groups with laryngeal and vocal alterations.

The inclusion of effect sizes deepened the interpretation of the results. It was observed that, for several measures, the differences between the groups not only reached statistical significance but also exhibited moderate to large magnitudes, especially for shimmer, HNR, HF noise, and CPPS in the connected speech task. These findings corroborate previous evidence that measures related to glottal noise, signal periodicity, and source stability are particularly sensitive to discriminating voices with laryngeal alterations or with greater vocal deviation^(41,43)^.

On the other hand, some measures had small effect sizes, reflecting differences of small magnitude and reinforcing the importance of interpreting statistical significance in conjunction with practical relevance.

In general, the joint analysis of hypothesis tests and effect sizes suggested that the acoustic measures evaluated capture distinct dimensions of vocal deviation, and that those related to noise and HNR stand out as more robust indicators of the vocal conditions studied^(43,44)^.

The results of this study indicated that, overall, the acoustic measures analyzed behaved similarly in the presence of laryngeal alterations and vocal deviations, suggesting that both types of impairment influence the parameters of stability and quality of the vocal signal equivalently.

The clinical group included different types of laryngeal alterations (nodules, polyps, cysts, and glottal chinks), reflecting the variability found in clinical practice. This heterogeneity can influence some acoustic patterns, since different lesions present their own vibratory behaviors. However, considering the study objective, this variability was treated as an inherent characteristic of the clinical group and represents a recognized limitation, typical of observational studies.

It is recommended that future research be developed with larger and more balanced samples, encompassing the diversity of laryngeal and vocal diagnoses, to broaden the external validity for a more in-depth understanding of the results obtained in this study.

It is important to emphasize that acoustic measures alone are not always sufficient to adequately describe the laryngeal condition or the vocal quality. An individual may present a measurement value within what is considered normal but still have some alteration that was not detected because the measurement was not sensitive enough or because the person made some adaptation. Nevertheless, it is essential to understand the information provided by each acoustic measure in the analysis of vocal production and in the characterization of vocal quality, as well as to identify the context in which each parameter performs best.

The applicability of multiparametric acoustic instruments is a relevant advance in human voice assessment, since the integration of various acoustic measures allows for a more comprehensive and accurate characterization of vocal properties. Moreover, this approach favors efficient analysis in different laryngeal and vocal production contexts, overcoming the limitations inherent to single-parameter analyses^(45)^.

## CONCLUSION

Women with laryngeal and vocal alterations had more deviated values ​​in the investigated acoustic measures, with a significant reduction in GNE, HNR, CPPS (speech), and HF noise, and an increase in jitter, shimmer, and spectral tilt values, indicating greater instability and the presence of noise in the vocal signal. Overall, the investigated acoustic measures differentiated between women with and without laryngeal and vocal alterations. The results indicate that the acoustic measures responded similarly to the presence of laryngeal alterations and vocal deviations, reinforcing the combined influence of structural and functional factors on the quality of the vocal signal.
