# Supplementary material for: Spectral, cepstral, and short-term acoustic measures in women with and without laryngeal and vocal alterations
Source: Codas. 2026 Jun 15;38(4):e20250344. doi: 10.1590/2317-1782/e20250344en (PMC13322492; doi:10.1590/2317-1782/e20250344en)
Supplement: Translation

# Translation: Medidas acústicas espectrais, cepstrais e de curto prazo em mulheres com e sem alterações laríngeas e vocais

**DOI:** 10.1590/2317-1782/e20250344pt

**Authors:** Amanda Mariane Paulino Gualberto, Fabiana Andrade Penido, Ana Cristina Côrtes Gama

**Affiliations:** 1 Curso de graduação em Fonoaudiologia, Faculdade de Medicina, Universidade Federal de Minas Gerais – UFMG - Belo Horizonte (MG), Brasil.; 2 Superintendência Central de Perícia Médica e Saúde Ocupacional do Estado de Minas Gerais - Belo Horizonte (MG), Brasil.; 3 Departamento de Fonoaudiologia, Universidade Federal de Minas Gerais – UFMG - Belo Horizonte (MG), Brasil.

**Keywords:** Análise Acústica da Fala, Voz, Alterações Laríngeas, Distúrbios da Voz, Qualidade da Voz

## Abstract

**Objetivo:**

Comparar medidas acústicas da voz de mulheres com e sem alterações laríngeas e vocais.

**Métodos:**

Estudo observacional analítico transversal com 69 professoras, divididas em quatro grupos: 21 com alterações laríngeas (GCA), 48 sem alterações (GSA), 45 com desvios vocais (GCD) e 24 sem desvios (GSD). Os grupos foram definidos pela avaliação otorrinolaringológica (videolaringoscopia ou videolaringoestroboscopia) e pela avaliação perceptivo-auditiva do grau de disfonia. Foram excluídas participantes fumantes, grávidas, em período menstrual, com alergias ou gripes. Amostras de voz e fala (3 segundos centrais da vogal [a] e contagem de 1 a 11) foram gravadas e editadas no Praat. As medidas acústicas Jitter, Shimmer, Harmonic Noise Ratio (HNR), Glottal to Noise Excitation (GNE), Smoothed Cepstral Peak Prominence (CPPS), Spectral tilt, High Frequency Noise (HF noise) e a diferença entre o primeiro e segundo harmônico (H1-H2) foram extraídas no VOXplot. A análise estatística foi realizada por meio do teste T, Mann-Whitney e Hedges’ g.

**Resultados:**

Mulheres com alterações laríngeas e vocais apresentaram valores menores de GNE, HNR, CPPS, H1-H2 e HF noise e valores maiores de Jitter, Shimmer e Spectral tilt. O CPPS, o Spectral tilt (vogal) e o H1–H2 (vogal e fala) não diferenciaram os grupos com e sem alterações laríngeas. O CPPS (vogal) e o Spectral tilt (vogal e fala) também não diferenciaram os grupos com e sem desvio vocal.

**Conclusão:**

As medidas acústicas responderam de forma semelhante à presença de alterações laríngeas e vocais, reforçando a influência conjunta de fatores estruturais e funcionais na qualidade do sinal vocal.

## INTRODUÇÃO

A voz humana é uma ferramenta primordial para comunicação, que reflete características individuais e de personalidade do emissor^(1)^. Além disso, a voz desempenha um papel crucial na qualidade da comunicação, influenciando a forma como uma mensagem é percebida e compreendida pelo ouvinte^(2,3)^. No entanto, a voz pode ser afetada por uma variedade de fatores, incluindo alterações anatômicas e fisiológicas na laringe, que podem resultar em diferenças notáveis em suas propriedades acústicas^(2)^. Entender as características acústicas da voz é essencial para a avaliação da qualidade vocal^(4)^.

Existe uma variedade de metodologias para a avaliação da voz que inclui, análise acústica, avaliação perceptivo-auditiva, avaliação aerodinâmica, exame visual laríngeo e autoavaliação vocal^(5)^. Cada uma dessas abordagens avaliativas desempenha um papel importante na avaliação multidimensional vocal, atribuindo uma visão abrangente das características da produção vocal^(6)^. A teoria mioelástica aerodinâmica é a mais aceita para explicar o processo de fonação e fornece informações importantes sobre o mecanismo vibratório das pregas vocais. Isso se torna especialmente relevante devido à associação de muitos distúrbios vocais a padrões anormais de oscilação das pregas vocais^(7)^.

Avaliar a qualidade de uma voz apresenta um desafio significativo, pois o julgamento subjetivo, baseado na percepção auditiva e considerado padrão-ouro, é influenciado por uma variedade de fatores, abrangendo desde elementos socioeconômicos e culturais até gostos pessoais, apesar de sua grande importância e utilização na clínica vocal^(1)^. Neste contexto, a avaliação acústica da voz vem para complementar a avaliação perceptivo-auditiva e ganhou destaque na clínica vocal devido à sua natureza não invasiva e objetiva^(5)^. Isso se evidencia com o contínuo desenvolvimento de diversos parâmetros acústicos, cujo propósito é mensurar o sinal vocal, e fornecer informações clínicas relevantes na avaliação das disfonias^(6)^.

A literatura^(8,9)^ descreve diversas medidas acústicas como: **1) os espectros e coeficientes obtidos por predição linear e de Fourier** ^(9,10)^ onde se encaixam as medidas cepstrais, *Cepstral Peak Prominence* (CPP) e *Smoothed Cepstral Peak Prominence* (CPPS), que evidenciam o destaque dos harmônicos em relação ao ruído presente no sinal de voz^(8)^; o *Spectral tilt,* caracterizado como o declínio espectral obtido a partir do espectro médio de longo termo (*Long-Term Average Spectrum* - LTAS) do som vocal irradiado pelos lábios, refletindo tanto as propriedades da fonte glótica quanto as características formânticas do trato vocal^(11)^; o *High Frequency Noise* (HF *noise*), que mensura a diferença entre a energia acústica relativa entre 0 a 6KHz e 6 a 10KHz^(11)^; o H1-H2, que analisa a diferença na amplitude do primeiro harmônico em relação ao segundo^(12)^; o *Harmonic Noise Ratio* (HNR), que representa a relação entre a energia periódica e a energia do ruído^(13)^; e o *Glottal to Noise Excitation Ratio* (GNE), que quantifica o ruído aditivo presente no sinal de voz^(14)^; **2) as medidas de perturbação de amplitude a curto prazo** ^(15)^, que quantificam variações ciclo a ciclo na amplitude do sinal de voz, como o *Shimmer* **3) as medidas de perturbação de frequência a curto prazo** ^(15)^, que quantificam variações ciclo a ciclo na frequência fundamental do sinal de voz, como o *Jitter*.

Essas medidas acústicas desempenham um papel fundamental na caracterização vocal, fornecendo informações objetivas e robustas, que podem ser utilizadas para avaliar diferentes quadros de disfonia e monitorar a evolução do tratamento vocal^(16)^.

Compreender como as alterações laríngeas afetam as características acústicas da voz é crucial para discernir as nuances vocais em contextos saudáveis e em quadros de disfonia, uma vez que essas alterações podem gerar uma falta de harmonia vocal, impedir a produção natural da voz, bem como alterar a qualidade da emissão vocal^(1,17)^. A aplicação confiável e válida da análise acústica objetiva na pesquisa ou na prática clínica requer atenção a requisitos específicos, tais como a qualidade da placa de som, tipo de microfone, programa de análise acústica e condições de exame, a fim de garantir uma análise vocal de elevada precisão e confiabilidade^(18)^, além da possibilidade de utilização de diferentes parâmetros acústicos que avaliam os sinais vocais nos domínios do tempo, frequência, amplitude e que frequência^(18)^.

Outro aspecto a se considerar é que a análise acústica centrada na avaliação de vogais sustentadas, tem sido uma prática comum tanto na pesquisa quanto na prática clínica. No entanto, a literatura ressalta a importância de enriquecer essa abordagem, incorporando não apenas a tarefa de vogais sustentadas, mas também a análise de fala encadeada^(9)^, quando possível. Tal expansão é fundamentada na crescente compreensão de que combinar a análise acústica em ambas as tarefas vocais contribui significativamente para a validade ecológica desse tipo de investigação vocal^(17)^. A inclusão de tarefas de fala adiciona um elemento mais realista à avaliação, refletindo de maneira mais fiel às condições naturais de uso da voz. Isso, por sua vez, proporciona estimativas mais robustas e abrangentes, aumentando a precisão diagnóstica dos parâmetros analisados^(9,17)^.

Nesse contexto, o objetivo da presente pesquisa foi comparar diferentes medidas acústicas nas tarefas de vogal sustentada e fala encadeada, da voz de mulheres com e sem alterações laríngeas e vocais. Por meio dessa análise comparativa, a pesquisa almeja enriquecer o conhecimento sobre as características acústicas de vozes femininas em diferentes contextos laríngeos e vocais, com alteração e sem alteração e, assim, contribuir para aprimorar a avaliação e o tratamento das disfonias.

Considerando a importância da voz como uma ferramenta fundamental em diversas profissões, desde o ensino até o canto, e o fato de que as alterações laríngeas podem exercer um impacto negativo na qualidade vocal e na vida profissional e pessoal das mulheres disfônicas^(19)^, torna-se imperativo compreender as variações acústicas associadas a esses quadros laríngeos e vocais. Essa compreensão é crucial para aprimorar a avaliação e o tratamento das disfonias na clínica vocal^(20)^.

## MÉTODO

Trata-se de um estudo observacional analítico transversal aprovado pelo comitê de ética em pesquisa da Universidade Federal de Minas Gerais (UFMG) sob o parecer 39351920.2.0000.5149.

Todas as participantes do estudo foram esclarecidas quanto aos objetivos e aos procedimentos da pesquisa e assinaram o Termo de Consentimento Livre e Esclarecido (TCLE).

Participaram do estudo 69 mulheres, professoras da rede estadual de Minas Gerais, com idades entre 22 e 44 anos (média= 36,81; DP= 6,64), divididas em dois grupos: 21 mulheres com alteração laríngea (GCA) e 48 mulheres sem alteração laríngea (GSA). A definição desses grupos foi baseada na avaliação otorrinolaringológica, realizada por meio da videolaringoscopia ou videolaringoestroboscopia.

As professoras foram submetidas à avaliação fonoaudiológica pericial da voz, na Superintendência Central de Perícia Médica e Saúde Ocupacional (SCPMSO) do Estado de Minas Gerais e apresentaram exame laríngeo, realizado por otorrinolaringologista de livre escolha.

O tamanho amostral corresponde ao total de professoras que atenderam simultaneamente aos critérios clínicos, vocais e laríngeos estabelecidos, constituindo uma amostra de conveniência, característica comum em delineamentos observacionais.

O critério de inclusão para o GSA foi o de não apresentar alterações laríngeas e de coaptação glótica, descritas pelos otorrinolaringologistas no laudo do exame laríngeo. Foram incluídas nesse grupo, as professoras que apresentaram a descrição de coaptação glótica completa ou presença de fenda triangular posterior, considerada fisiológica em mulheres^(21)^.

Para o GCA, foram incluídas professoras cujo laudo otorrinolaringológico indicou a presença de insuficiência na coaptação glótica e alterações nas bordas das pregas vocais. Os exames foram realizados por diferentes médicos otorrinolaringologistas. As gravações foram posteriormente analisadas, de forma independente, por duas fonoaudiólogas especialistas em voz, com o objetivo de verificar a presença das alterações descritas no laudo. Foram incluídos no estudo apenas os casos em que houve concordância entre o diagnóstico otorrinolaringológico e a análise consensual das fonoaudiólogas, definida quando ambas as profissionais verificaram as alterações laríngeas nas gravações. Das 23 gravações analisadas, 2 foram excluídas por não apresentarem essa concordância, permanecendo na amostra final apenas os casos com avaliação consensual.

Os critérios de exclusão para ambos os grupos do estudo foram ser fumante, ser mulher grávida ou estar no período menstrual e estar em processo alérgico ou em quadro gripal.

As vozes das 69 professoras foram gravadas em uma sala silenciosa com um ruído ambiental abaixo de 40dB NPS, medido por meio de um decibelímetro marca DEC – modelo 5010, na SCPMSO.

As amostras de voz e de fala foram gravadas em um computador da marca Apple, modelo MacBook Air, com placa de som profissional utilizando um microfone condensador unidirecional, modelo adaptador *Andrea Pureaudio USB e microfone condensador unidirecional Karsect HT-9*, montado na cabeça, a uma distância de 10cm entre o microfone e a comissura labial das participantes. As amostras foram digitalizadas com taxa de amostragem de 44,1kHz e resolução de amplitude de 16bits, no formato .wav, no *software* Praat (versão 6.1.47)^(5)^.

Para a extração das medidas acústicas, as professoras ficaram de pé e foram instruídas a realizar as seguintes tarefas: emissão sustentada da vogal [a:] e contagem de 1 a 20, em frequência e intensidade habituais.

Após a gravação das amostras, os arquivos de voz e de fala foram editados no *software* Praat versão 6.1.47. Selecionou-se os 3s centrais da vogal [a:], manualmente, em frequência e intensidade habituais, por corresponderem à porção mais estável do sinal vocal. Além disso, utilizou-se a contagem de números de 1 a 11, como tarefa de fala encadeada, por tratar-se de uma tarefa amplamente empregada na prática clínica fonoaudiológica brasileira e por apresentar melhor correlação entre medidas perceptivas e acústicas^(18)^ (Figura 1).

**Figura 1:** Telas do Praat referente a seleção dos 3 segundos centrais da vogal [a] (acima) e da contagem de 1 a 11 (abaixo)

Os arquivos de voz e de fala, gravados e editados no Praat, foram nomeados como sv (*sustained vowel*) e cs (*continuous speech*), respectivamente e carregados no VOXplot versão 2.5.0^(11)^, para extração das medidas acústicas (Figura 2). O *software* VOXplot calcula as medidas acústicas das tarefas vocálica e de contagem, evidencia o oscilograma e o espectrograma de banda estreita da vogal [a], além de mostrar um gráfico que ilustra os valores de seis dimensões acústicas, utilizadas para avaliar a rouquidão e a soprosidade na voz, a saber HNR, *Jitter* (ppq5), GNE, CPPS, Acoustic Voice Quality Index (AVQI) e Acoustic Breathiness Index (ABI)^(22)^ (Figura 2).

**Figura 2:** Tela de análise da qualidade vocal e das medidas acústicas do VOXplot versão 2.5.0

As medidas acústicas de *Jitter* (%), *Shimmer* (%), HNR (dB) e GNE foram obtidas por meio da vogal, enquanto o *Spectral tilt* - declínio espectral (dB), HF *noise* (dB), H1-H2 (dB) e CPPS (dB), foram obtidos por meio da tarefa vocálica e da contagem de números.

Com o intuito de caracterizar os grupos do ponto de vista da qualidade vocal, a avaliação perceptivo-auditiva das amostras de voz e de fala foi realizada de modo independente por três fonoaudiólogas, com mais de 10 anos de experiência nesse tipo de análise. As amostras foram classificadas de acordo com a escala GRBASI^(23)^, utilizando o parâmetro G, referente ao grau geral da qualidade vocal. A GRBASI é uma escala likert de 4 pontos, sendo 0= ausência de alteração vocal, 1= alteração vocal leve, 2= alteração vocal moderada e 3= alteração vocal intensa.

A análise estatística dos dados foi realizada por meio do programa estatístico MINITAB versão 17. Primeiramente foi realizada uma análise descritiva dos dados com medidas de tendência central e dispersão. Posteriormente, foi utilizado o teste de Anderson-Darling para verificar a normalidade da amostra. Para comparação das medidas acústicas HNR (dB), CPPS (dB), *Spectral tilt*, HF *noise* (dB) e H1-H2 (dB), entre os grupos GCA e GSA utilizou-se o teste T e para as medidas de *Jitter*, *Shimmer* e GNE o teste não paramétrico Mann-Whitney. Considerou-se o nível de confiança de 95%.

Foi calculado o tamanho de efeito para cada medida acústica por meio do Hedges’ g, medida adequada para comparar dois grupos independentes, com tamanhos amostrais moderados e distinta por corrigir a estimativa do Cohen’s d para amostras pequenas. Os tamanhos de efeito foram interpretados de acordo com os critérios convencionais: pequeno (≈ 0,2), moderado (≈ 0,5) e grande (≥ 0,8)^(24)^.

## RESULTADOS

O GCA foi composto por 21 mulheres, que receberam o diagnóstico otorrinolaringológico de nódulos (n= 16; 76,2%), pólipo (n= 2; 9,5%), cisto (n= 1; 4,8%) e fenda glótica em ampulheta e triangular médio-posterior (n= 2; 9,5%).

Em relação ao G da escala GRBASI, as três fonoaudiólogas peritas classificaram as amostras de voz e de fala dos grupos com e sem alteração laríngea. De acordo com a classificação de pelo menos duas das três fonoaudiólogas peritas, o GSA foi composto por 24 (50%) mulheres com qualidade vocal neutra (G0) e 24 (50%) foram classificadas com grau geral de desvio leve (G1). Já o GCA foi composto por 10 (47,61%) professoras com alterações vocais leves, 7 (33,33%) com alterações vocais moderadas e 4 (19,06%) com alterações vocais intensas.

Na Tabela 1 podem ser observadas as médias, o desvio padrão e a comparação das medidas acústicas, entre o GSA e GCA.

**Tabela 1:** Comparação das medidas acústicas entre os grupos de mulheres sem e com alteração laríngea

**MEDIDAS ACÚSTICAS**	**GRUPO**	**MÉDIA**	**DP**	**p-valor**	**TAMANHO DO EFEITO**
***Jitter* (%) (sv)**	GSA	0,35	0,21	**0,017#**	-0,67
	GCA	0,54	0,36		
***Shimmer* (%) (sv)**	GSA	2,66	1,55	**< 0,001** ^#^	-1,08
	GCA	4,71	2,26		
**HNR (dB) (sv)**	GSA	24,66	4,26	< 0,000*	1,21
	GCA	19,44	4,26		
**GNE (sv)**	GSA	0,93	0,07	**0,0094#**	0,63
	GCA	0,88	0,09		
**CPPS (sv)**	GSA	15,61	2,23	0,063*	0,53
	GCA	14,29	2,75		
**CPPS (cs)**	GSA	12,87	1,38	0,002*	1,01
	GCA	11,11	2,12		
**H1-H2 (dB) (sv)**	GSA	4,33	5,46	0,277*	0,29
	GCA	2,7	5,71		
**H1-H2 (dB) (cs)**	GSA	1,74	2,82	0,062*	0,57
	GCA	-0,45	4,81		
**Spectral *tilt* (dB) (sv)**	GSA	-10,59	1,17	0,26*	-0,31
	GCA	-10,2	1,35		
**Spectral *tilt* (dB) (cs)**	GSA	-11,58	0,99	0,009*	-0,8
	GCA	-10,61	1,44		
**HF *Noise* (dB) (sv)**	GSA	2,19	0,2	**< 0,000** ^*^	1,09
	GCA	1,96	0,22		
**HF *Noise* (dB) (cs)**	GSA	2,26	0,19	0,005*	0,85
	GCA	2,07	0,26		

* t-test;

# Mann-Whitney Test

**Legenda:** HNR: *Harmonic Noise Ratio*; GNE: *Glottal to Noise Excitation* **;** CPPS: *Smoothed Cepstral Peak Prominence*; H1-H2: *Amplitude Difference* H1-H2; *Spectral tilt: Spectral Decline*; HF *Noise*: *High Frequency Noise*; sv: *sustained vowel;* cs: *continuous speech*; dB: decibel; Hz: Hertz; DP: desvio padrão; GSA: grupo sem alteração laríngea; GCA: grupo com alteração laríngea

A análise comparativa entre os grupos evidenciou que mulheres com presença de alterações laríngeas (GCA) apresentam menores valores, com diferença estatisticamente significativa, nos parâmetros acústicos GNE (vogal), HNR (vogal), CPPS (fala) e HF *noise* (vogal e fala). Observou-se também valores maiores das medidas acústicas *Jitter* (vogal)*, Shimmer* (vogal) e *Spectral tilt* (fala) no GCA, com diferença significativa entre os grupos. O GCA apresentou valores menores de H1-H2 (vogal e fala) e CPPS (vogal), sem diferença significativa entre os grupos. Para o *Spectral tilt*, na tarefa de vogal, o GCA apresentou valor maior na comparação com o GSA, sem diferença significativa.

Observou-se tamanhos de efeito grandes para *Shimmer* e HNR (vogal), CPPS e *Spectral tilt* (fala) e HF *noise* (vogal e fala). Efeitos moderados foram encontrados para *Jitter*, GNE e CPPS (vogal) e H1-H2 (fala). Esses achados indicam que, além de estatisticamente significativas, várias dessas diferenças apresentam magnitude suficiente para ser considerada clinicamente relevante.

A tabela 2 apresenta a comparação das medidas acústicas entre os grupos de mulheres sem desvios vocais (GSD) (n= 24) e aquelas com desvios vocais (GCD) (n=45), composto por mulheres com alterações vocais de graus leve (n= 34), moderado (n= 7) e intenso (n= 4).

**Tabela 2:** Comparação das medidas acústicas entre os grupos de mulheres sem e com desvio vocal

**MEDIDAS ACÚSTICAS**	**GRUPO**	**MÉDIA**	**DP**	**p-valor**	**TAMANHO DO EFEITO**
***Jitter* (%) (sv)**	GSD	0,27	0,09	**0,0017** ^#^	-0,98
	GCD	0,48	0,31		
***Shimmer* (%) (sv)**	GSD	2,39	1,54	**0,0025#**	-0,76
	GCD	3,76	2,09		
**HNR (dB) (sv)**	GSD	26,12	4,56	< 0,000*	1,04
	GCD	21,44	4,24		
**GNE (sv)**	GSD	0,94	0,04	**0,03#**	0,6
	GCD	0,9	0,09		
**CPPS (sv)**	GSD	15,86	2,34	0,104^*^	0,41
	GCD	14,86	2,46		
**CPPS (cs)**	GSD	12,99	1,45	0,018*	0,6
	GCD	11,99	1,91		
**H1-H2 (dB) (sv)**	GSD	5,99	4,13	0,009*	0,66
	GCD	2,68	5,9		
**H1-H2 (dB) (cs)**	GSD	2,52	2,78	0,008*	0,67
	GCD	0,3	3,85		
**Spectral *tilt* (dB) (sv)**	GSD	-10,65	1,02	0,358*	-0,23
	GCD	-10,38	1,33		
**Spectral *tilt* (dB) (cs)**	GSD	-11,61	1,11	0,103*	-0,41
	GCD	-11,12	1,26		
**HF *Noise* (dB) (sv)**	GSD	2,23	0,19	0,002*	0,81
	GCD	2,06	0,23		
**HF *Noise* (dB) (cs)**	GSD	2,29	0,19	0,016*	0,62
	GCD	2,16	0,23		

* t-test;

# Mann-Whitney Test

**Legenda:** HNR: *Harmonic Noise Ratio*; GNE: *Glottal to Noise Excitation* **;** CPPS: *Smoothed Cepstral Peak Prominence*; H1-H2: *Amplitude Difference* H1-H2; *Spectral tilt: Spectral Decline*; HF *Noise*: *High Frequency Noise*; sv: *sustained vowel;* cs: *continuous speech*; dB: decibel; Hz: Hertz; DP: desvio padrão; GSD: grupo sem desvio vocal; GCD: grupo com desvio vocal

Diferenças significativas entre os grupos foram observadas para todas as medidas acústicas, com exceção do CPPS (vogal) e do *Spectral tilt* (vogal e fala).

O grupo de mulheres com desvios vocais apresentou valores significativamente menores para o GNE (vogal), HNR (vogal), CPPS (fala) e HF *noise* (vogal e fala), padrão semelhante ao observado na comparação entre os grupos com e sem alterações laríngeas. A medida H1-H2 também apresentou diferença estatisticamente significativa entre os grupos, nas tarefas de vogal e fala, com valores menores observados no grupo com desvios vocais. Tal fato não foi verificado na comparação entre os grupos com e sem alterações laríngeas.

Valores maiores foram observados para as medidas de *Jitter* e *Shimmer* no grupo com desvios vocais, com diferença estatisticamente significativa.

Observou-se tamanhos de efeitos grandes para *Jitter*, HNR e HF *noise* (vogal), e efeitos moderados para *Shimmer* e GNE (vogal), CPPS (fala) e H1-H2 em ambas as tarefas. Parâmetros espectrais de inclinação (*Spectral tilt*) apresentaram efeitos pequenos, mesmo quando acompanhados de diferenças estatisticamente significativas.

## DISCUSSÃO

A voz é uma característica fundamental na comunicação humana e reflete a saúde e a funcionalidade da laringe^(3)^. Distúrbios laríngeos podem gerar um impacto significativo na qualidade vocal, sendo necessárias ferramentas de diagnóstico precisas e sensíveis para a sua detecção e monitoramento^(1)^.

A análise acústica da voz surge como um recurso valioso nesse cenário, pois permite uma avaliação objetiva e quantitativa das características vocais^(9)^. Nesta pesquisa, buscou-se investigar o comportamento das medidas acústicas que trazem informações em relação à fonte glótica, tais como a *Jitter* (%)*, Shimmer* (%)*,* HNR*,* GNE, *Spectral tilt,* CPPS e H1-H2, nas tarefas de vogal sustentada e fala encadeada, da voz de mulheres com e sem alterações laríngeas e vocais.

No que diz respeito às medidas de perturbação de frequência e de amplitude, *Jitter* (%) e *Shimmer* (%), verificou-se que tanto o grupo de mulheres com alterações laríngeas quanto o grupo com desvios vocais apresentaram valores maiores dessas medidas na vogal [a] quando comparado com os grupos sem alterações laríngeas e sem desvios vocais, conforme esperado (tabelas 1 e 2). Além disso, essas medidas apresentaram diferenças significativas entre os grupos com e sem alterações laríngeas e vocais. Tal fato, indica que as alterações laríngeas apresentadas pelas mulheres do GCA interferiram na periodicidade da vibração de frequência e amplitude das pregas vocais a curto prazo, gerando um quadro de alteração na qualidade vocal.

Existem várias formas para obtenção do *Jitter* e do *Shimmer*, sendo que nesse estudo, analisou-se os valores de *Jitter* e *Shimmer* local (%). O *Jitter local* (%) relaciona-se à diferença média absoluta entre períodos sucessivos, dividida pelo período médio. O *Shimmer* local (%) refere-se a diferença média absoluta entre as amplitudes de períodos sucessivos dividido pela amplitude média^(13)^.

Estudos mostram que o *Shimmer* altera-se, principalmente, nas situações de redução da resistência glótica (por exemplo: coaptação glótica incompleta) e também é influenciado por lesões de massa, mesmo as de tamanho reduzido^(1,4)^. Já o *Jitter* altera-se, principalmente, com a falta de controle da vibração das pregas vocais^(25)^.

Uma pesquisa realizada com mulheres com queixa vocal, onde foram extraídas as medidas de *Jitter* e *Shimmer*, além de outras medidas acústicas verificou o aumento dos valores dessas medidas diante da piora da qualidade vocal^(26)^. Neste estudo, conforme mencionado, o desvio vocal esteve associado ao aumento dos valores dessas medidas, assim como a presença de alterações laríngeas.

O *Shimmer* também apresentou diferença significativa em outro estudo desenvolvido em mulheres com e sem nódulos vocais^(10)^. Os valores de *Jitter* e *Shimmer* obtidos para as mulheres com nódulos vocais foram 0,87% e 6,91%, enquanto para mulheres sem nódulos foram 0,28% e 3,31%, com a vogal [a]. Da mesma forma, no presente estudo, os valores de *Jitter* e *Shimmer* foram menores no GSA quando comparados com o GCA (GCA: *Jitter*= 0,54%; *Shimmer*= 4,71%; GSA: *Jitter*= 0,35%; *Shimme*r= 2,66%), sendo que o GCA apresentou valores desviados. No*software*utilizado, VOXplot, os valores de referência para os parâmetros de*Jitter (%)* e *Shimmer (%)* para vozes saudáveis são <0,5% e <3%, respectivamente^(11)^.

Os resultados obtidos no presente estudo para as medidas *Jitter* e *Shimmer* também estão em consonância com um estudo que verificou diferença significativa de ambas as medidas em sinais de voz controle e sinais provenientes de alterações laríngeas, tais como pólipos nas pregas vocais^(27)^.

Em relação ao HNR, outra medida acústica considerada de curto prazo, verificou-se diferenças significativas entre os grupos, com e sem alterações laríngeas e vocais. O HNR indica a relação entre os componentes periódico (correspondente à vibração das pregas vocais) e aperiódico (ruído glótico) do sinal de voz, fornecendo informações relacionadas à eficiência do processo de fonação. Dessa forma, quanto menor for o componente harmônico, maior será o componente de ruído^(28)^. Os achados do presente estudo, indicam que as alterações laríngeas apresentadas pelas mulheres do GCA, impactaram na eficiência do processo de fonação. Em geral, lesões em borda de pregas vocais resultam em coaptação glótica incompleta, com escape de ar e turbulência do fluxo aéreo durante a produção vocal^(29)^. Assim, era esperado que tanto as mulheres do GCA quanto as do GCD apresentassem valores menores do HNR do que as mulheres do GSA e GSD, conforme foi verificado (HNR: GCA= 19,44 e GSA= 24,66; GCD= 21,44 e GSD= 26,12). Quanto maior o valor dessa medida acústica, menor é a presença de ruído no sinal acústico.

Esses achados são coerentes com estudos que investigaram o HNR para diferenciar vozes com e sem desvios^(30,31)^. Pesquisadores também encontraram forte correlação entre essa medida e o parâmetro de rouquidão na qualidade vocal^(11,32)^.

Considerada uma medida de harmonicidade, o GNE pode ser utilizado para quantificar o ruído aditivo presente no sinal de voz e é uma medida robusta para discriminar a soprosidade da rouquidão^(11)^ e a intensidade do desvio vocal^(26)^. Por meio dessa medida é possível estimar a quantidade de energia proveniente da vibração das pregas vocais e da turbulência provocada pelo desvio vocal, quando presente. Valores mais altos do GNE indicam uma maior proporção de energia glótica em relação à energia de ruído do sinal acústico^(33)^. Ao analisar o GNE verificou-se que os grupos sem alterações laríngeas e vocais (GSA e GSD) apresentaram valores significativamente maiores em comparação aos grupos com alterações (GCA e GCD), conforme demonstrado nas medianas apresentadas nas tabelas 1 e 2. Valores maiores de GNE para mulheres sem nódulos vocais também foram verificados em um estudo desenvolvido em mulheres com e sem nódulos vocais^(10)^ (GNE= 0,63 e 0,82; respectivamente).

Um estudo que investigou os parâmetros acústicos mais eficazes para estimar a rouquidão e a soprosidade na qualidade vocal, utilizando como tarefa a emissão da vogal [a] e o *software* VOXplot, verificou que valores do GNE abaixo de 0,91 tendem a sinalizar a rouquidão e valores abaixo de 0,89 indicam a presença de soprosidade^(11)^. O GNE e o CPPS foram consideradas as melhores medidas para detectar a soprosidade na qualidade vocal^(11)^. No presente estudo, observou-se que os grupos sem alterações laríngeas e vocais apresentaram valores do GNE acima dos pontos de corte verificados no estudo supramencionado (GNE: GSA= 0,93 e GSD= 0,94), indicando eficiência glótica adequada nesses grupos.

Ao investigar a medida *cepstral*, medida possível de ser extraída tanto na vogal sustentada quanto na tarefa de fala encadeada, observou-se que o CPPS apresentou diferenças significativas entre os grupos de mulheres com e sem alterações laríngeas e vocais, apenas para a amostra de fala, com valores maiores de CPPS para o GSA e o GSD, conforme esperado (CPPS: GSA= 12,87 e GCA= 11,11; GSD= 12,99 e GCD= 11,99). Tal fato, indica que as mulheres do GSA e do GSD apresentaram uma maior estrutura harmônica quando comparadas com as mulheres dos grupos com alterações laríngeas e vocais.

Em consonância com a presente pesquisa, um estudo que investigou a medida *cepstral* na vogal [a], em indivíduos com nódulos constatou um valor menor dessa medida na presença da lesão laríngea^(34)^. Outro estudo verificou que indivíduos com vozes sem alterações apresentaram valores mais altos do CPPS, em decorrência de uma estrutura harmônica bem definida. Em contrapartida, indivíduos com desvios vocais apresentaram valores menores dessa medida^(8)^.

O valor do CPPS pode variar de acordo com a tarefa. Em geral, o valor do CPPS obtido para a vogal sustentada é maior quando comparado com seu valor na tarefa de fala, devido a maior estabilidade na fonação durante a execução da vogal^(35)^. Nesse estudo, observou-se essa tendência, porém não se verificou diferenças significativas entre os grupos para a tarefa de emissão da vogal [a]. Esse achado está em consonância com um estudo que investigou as medidas acústicas e perceptivo-auditivas em mulheres com e sem nódulos vocais^(10)^. Tal fato, pode estar relacionado à tarefa, uma vez que o CPPS extraído da tarefa de fala apresenta maior poder discriminatório entre voz normal e alterada, quando comparado ao CPPS da vogal^(35)^.

As medidas a curto prazo, *Jitter*, *Shimmer* e HNR, mostraram-se sensíveis à discriminação entre os grupos com e sem alterações laríngeas e vocais. A literatura revela que as medidas a curto prazo apresentam utilidade mais direcionada às alterações vocais de graus leves e moderados, uma vez que a extração de seus valores depende da comparação da estrutura harmônica ciclo a ciclo. Assim, para que o uso dessas medidas seja viável, as vozes não podem apresentar uma estrutura fortemente aperiódica^(28)^, fato verificado com a amostra de vozes do presente estudo, que foi composta por apenas 4 mulheres com desvio vocal intenso.

Na análise dos resultados obtidos para a medida H1-H2, que representa a diferença na amplitude do primeiro harmônico em relação ao segundo, observaram-se valores menores para o GCA e GCD, assim como verificado em estudos anteriores^(36,37)^. O primeiro harmônico se relaciona ao pico de amplitude da *fo* em decibéis (dB) (H1), enquanto o segundo harmônico refere-se ao pico de amplitude de duas vezes o valor da *fo* (H2). A relação entre essas medidas revela características fonatórias da voz. Além disso, o valor de H1-H2 está associado ao comprimento relativo da fase aberta da oscilação glótica^(38)^. Uma fase de fechamento glótico incompleto ou lento parece aumentar a diferença da amplitude entre H1 e H2. Dessa forma, em vozes soprosas, espera-se que a amplitude do primeiro harmônico seja mais alta em comparação com os outros harmônicos. Em contrapartida, quando o H2 é mais alto do que o H1 (produzindo um valor negativo), espera-se a presença de uma voz tensa ou uma voz com produção mais firme, com maior pressão subglótica. Diante de uma voz sem desvios, os valores de H1 e H2 são semelhantes^(39)^.

No presente estudo, os grupos sem alterações laríngeas e vocais apresentaram valores maiores do H1-H2, na vogal e na fala, quando comparado com o GCA e o GCD (tabelas 1 e 2). Além disso, verificou-se diferenças significativas nessa medida acústica somente na comparação dos grupos com e sem desvios vocais.

O fato do GCA e GCD apresentarem valores menores do H1-H2 em comparação aos grupos sem alterações (CSA e GSD) não foi reproduzido em dois estudos realizados no português brasileiro (PB), mas foi observado em uma investigação conduzida com mulheres portadoras de lesões laríngeas e sem desvios vocais^(40)^. Um dos estudos realizados no PB analisou o H1-H2 em mulheres com disfonia comportamental, com e sem alterações laríngeas^(41)^ e verificou valores médios maiores para o grupo com lesões (H1-H2= 3,64) em relação ao grupo sem lesões (H1-H2= 1,65). De forma semelhante, outro estudo desenvolvido com mulheres com e sem nódulos vocais identificou valores de H1-H2 maiores no grupo com lesão (H1-H2 = 7,42) do que no grupo controle (H1-H2 = 4,94)^(10)^.

Cabe ressaltar, que o H1-H2 é uma medida acústica que apresenta grande variabilidade na comparação intersujeitos^(42)^. No presente estudo e nos estudos mencionados para o PB^(10,41)^, essa variabilidade foi observada, evidenciada pela ampla dispersão dos valores de H1-H2 em torno das médias dos grupos. Pesquisas futuras, com uma amostra maior e com maior validade externa são necessárias para melhor compreensão destes resultados.

O *Spectral tilt* descreve a inclinação da reta de regressão no espectro LTAS (do inglês, *Long-Term Average Spectrum*)^(11)^. Quanto maior a inclinação da reta, menor o valor dessa medida. Quanto à comparação do *Spectral tilt*, obtidas nas amostras de vogal e de fala, observou-se diferença significativa apenas entre os grupos com e sem alterações laríngeas, nas amostras de fala. Além disso, verificou-se que o GSA e o GSD apresentaram valores menores dessa medida quando comparado com o GCA e GCD, indicando maior inclinação da reta, nas mulheres sem alterações laríngeas e vocais.

Os resultados deste estudo indicam que a presença de alterações laríngeas e vocais gerou um aumento no valor do *Spectral tilt* e, consequentemente menor inclinação da reta. Esse achado pode estar relacionado à adoção de mecanismos compensatórios e ao aumento da tensão durante a coaptação glótica nas mulheres do GCA, que majoritariamente apresentaram lesões nas bordas das pregas vocais.

Em um estudo que investigou as medidas de um índice acústico multiparamétrico, a saber, o Acoustic Voice Quality Index (AVQI), em indivíduos com e sem disfonia, secundária a diferentes condições patológicas, não verificou diferenças no *Spectral tilt* entre os grupos. Os pesquisadores concluíram que o uso dessa medida poderia não auxiliar na discriminação de condições patológicas quando utilizada de forma isolada^(11)^.

A literatura se mostra escassa em estudos que investigaram as medidas acústicas *Spectral tilt* e HF *noise*, de forma isolada, para diferenciar grupos com e sem alterações laríngeas e vocais. No que diz respeito ao HF *noise*, que mensura o nível relativo de ruído de alta frequência entre a energia de 0 a 6KHz e a energia de 6 a 10KHz^(11)^ observou-se que essa medida foi sensível tanto na vogal quanto na fala e revelou diferenças significativas entre os grupos com e sem alterações laríngeas e vocais (tabelas 1 e 2). O GCA e GCD apresentaram valores menores dessa medida para as duas tarefas investigadas, quando comparado com o GSA e GSD, conforme esperado. Tal fato, indica maior energia ruidosa em frequência altas do espectro, acima de 6KHz, na presença de alterações laríngeas e vocais.

Em um estudo que buscou comparar os resultados de validade diagnóstica de medidas acústicas isoladas da qualidade vocal que são mais fortes para a percepção da rouquidão e soprosidade, por meio da utilização do *software* VOXplot, verificou pontos de corte do HF *noise* (vogal) de 2,28 dB e 2,29 dB para a percepção da rouquidão e soprosidade^(11)^. No presente estudo, os valores dessa medida estão abaixo dos pontos de corte estabelecidos para a percepção de soprosidade e rouquidão, nos grupos com alterações laríngeas e vocais.

A inclusão dos tamanhos de efeito aprofundou a interpretação dos resultados obtidos. Observou-se que, para várias medidas, as diferenças entre os grupos não apenas atingiram significância estatística, mas também exibiram magnitudes moderadas a grandes, especialmente para *Shimmer*, HNR, HF *noise* e CPPS na tarefa de fala encadeada. Esses achados corroboram com evidências prévias de que medidas relacionadas ao ruído glótico, à periodicidade do sinal e à estabilidade da fonte são particularmente sensíveis para discriminar vozes com alterações laríngeas ou com maior desvio vocal^(41,43)^.

Por outro lado, algumas medidas apresentaram tamanhos de efeito pequenos, refletindo diferenças de pequena magnitude e reforçando a importância de interpretar a significância estatística em conjunto com a relevância prática.

De forma geral, a análise conjunta dos testes de hipótese e dos tamanhos de efeito sugeriu que as medidas acústicas avaliadas captam dimensões distintas do desvio vocal, e que aquelas relacionadas ao ruído e à relação harmônico-ruído se destacam como indicadores mais robustos das condições vocais estudadas^(43,44)^.

Os resultados do presente estudo indicaram que, no geral, as medidas acústicas analisadas apresentaram comportamento semelhante diante da presença de alterações laríngeas e de desvios vocais, sugerindo que ambos os tipos de comprometimento influenciam de maneira equivalente nos parâmetros de estabilidade e qualidade do sinal vocal.

O grupo clínico incluiu diferentes tipos de alterações laríngeas (nódulos, pólipos, cistos e fendas glóticas), o que reflete a variabilidade encontrada na prática clínica. Essa heterogeneidade pode influenciar alguns padrões acústicos, uma vez que distintas lesões apresentam comportamentos vibratórios próprios. Contudo, considerando o objetivo do estudo, essa variabilidade foi tratada como característica inerente ao grupo clínico e representa uma limitação reconhecida, típica de estudos observacionais.

Recomenda-se o desenvolvimento de pesquisas futuras com amostras mais amplas e equilibradas, que contemplem a diversidade de diagnósticos laríngeos e vocais, de modo a ampliar a validade externa para uma compreensão mais aprofundada dos resultados obtidos neste estudo.

É importante ressaltar que a utilização de medidas acústicas isoladas nem sempre é suficiente para descrever adequadamente a condição laríngea ou até mesmo a qualidade vocal. Um indivíduo pode apresentar um valor de uma medida dentro do que é considerado normal, mas ainda assim ter alguma alteração, que não foi detectada porque a medida não foi suficientemente sensível ou porque a própria pessoa realizou alguma adaptação. No entanto, é fundamental compreender as informações fornecidas por cada medida acústica na análise da produção vocal e na caracterização da qualidade vocal, assim como identificar os contextos em que cada parâmetro apresenta melhor desempenho.

A aplicabilidade de instrumentos acústicos multiparamétricos constitui um avanço relevante na avaliação da voz humana, pois a integração de diversas medidas acústicas permite uma caracterização mais abrangente e acurada das propriedades vocais. Além disso, essa abordagem favorece uma análise eficiente em diferentes contextos laríngeos e de produção vocal, superando as limitações inerentes às análises de monoparâmetros^(45)^.

## CONCLUSÃO

Mulheres com alterações laríngeas e vocais apresentaram valores mais desviados das medidas acústicas investigadas, com redução significativa do GNE, HNR, CPPS (fala) e HF *noise* e aumento nos valores do *Jitter, Shimmer* e *Spectral tilt*, indicando maior instabilidade e presença de ruído no sinal vocal. De modo geral, verificou-se que as medidas acústicas investigadas foram capazes de diferenciar as mulheres com e sem alterações laríngeas e vocais. Os resultados indicaram que as medidas acústicas responderam de forma semelhante à presença de alterações laríngeas e de desvios vocais, reforçando a influência conjunta de fatores estruturais e funcionais na qualidade do sinal vocal.
